# Associations between telomere attrition, genetic variants in telomere maintenance genes, and non-small cell lung cancer risk in the Jammu and Kashmir population of North India

**DOI:** 10.1186/s12885-023-11387-z

**Published:** 2023-09-18

**Authors:** Gh. Rasool Bhat, Rajeshwer Singh Jamwal, Itty Sethi, Amrita Bhat, Ruchi Shah, Sonali Verma, Minerva Sharma, Hana Q. Sadida, Sara K. Al-Marzooqi, Tariq Masoodi, Sameer Mirza, Mohammad Haris, Muzafar A. Macha, Ammira S. Alshabeeb Akil, Ajaz A. Bhat, Rakesh Kumar

**Affiliations:** 1 School of Biotechnology, Shri Mata Vaishno Devi University, Katra, Jammu and Kashmir 182320 India; 2 Institute of Human Genetics, University of Jammu, Jammu and Kashmir, 180001 India; 3 Department of Human Genetics-Precision Medicine in Diabetes, Obesity & Cancer Program, Sidra Medicine, 26999 Doha, Qatar; 4 Laboratory of Cancer Immunology and Genetics, Sidra Medicine, 26999 Doha, Qatar; 5 Department of Chemistry, College of Sciences, United Arab , Emirates University, 15551 Al-Ain, United Arab Emirates; 6 Center for Advanced Metabolic Imaging in Precision Medicine, Department of Radiology, Perelman School of Medicine, University of Pennsylvania, Philadelphia, USA; 7 Watson-Crick Centre for Molecular Medicine, Islamic University of Science and Technology, 192122, Jammu and Kashmir, India

**Keywords:** Biomarker, Gene variants, Telomere maintenance gene, Non-small cell lung cancer, Telomere length

## Abstract

**Background:**

Telomeres are repetitive DNA sequences located at the ends of chromosomes, playing a vital role in maintaining chromosomal integrity and stability. Dysregulation of telomeres has been implicated in the development of various cancers, including non-small cell lung cancer (NSCLC), which is the most common type of lung cancer. Genetic variations within telomere maintenance genes may influence the risk of developing NSCLC. The present study aimed to evaluate the genetic associations of select variants within telomere maintenance genes in a population from Jammu and Kashmir, North India, and to investigate the relationship between telomere length and NSCLC risk.

**Methods:**

We employed the cost-effective and high-throughput MassARRAY MALDI-TOF platform to assess the genetic associations of select variants within telomere maintenance genes in a population from Jammu and Kashmir, North India. Additionally, we used TaqMan genotyping to validate our results. Furthermore, we investigated telomere length variation and its relation to NSCLC risk in the same population using dual-labeled fluorescence-based qPCR.

**Results:**

Our findings revealed significant associations of TERT rs10069690 and POT1 rs10228682 with NSCLC risk (adjusted *p*-values = 0.019 and 0.002, respectively), while TERF2 rs251796 and rs2975843 showed no significant associations. The TaqMan genotyping validation further substantiated the associations of TERT rs10069690 and rs2242652 with NSCLC risk (adjusted *p*-values = 0.02 and 0.003, respectively). Our results also demonstrated significantly shorter telomere lengths in NSCLC patients compared to controls (*p* = 0.0004).

**Conclusion:**

This study highlights the crucial interplay between genetic variation in telomere maintenance genes, telomere attrition, and NSCLC risk in the Jammu and Kashmir population of North India. Our findings suggest that TERT and POT1 gene variants, along with telomere length, may serve as potential biomarkers and therapeutic targets for NSCLC in this population. Further research is warranted to elucidate the underlying mechanisms and to explore the potential clinical applications of these findings.

**Supplementary Information:**

The online version contains supplementary material available at 10.1186/s12885-023-11387-z.

## Introduction

Lung cancer, a leading cause of cancer-related mortality worldwide, is a multifactorial disease influenced by both genetic and non-genetic factors [1, 2]. The increasing prevalence of lung cancer in the Indian population, particularly in the Jammu and Kashmir (J&K) region of North India, necessitates the investigation of pathways involved in its etiology. Genome-wide association studies (GWAS) have uncovered numerous genetic variants in telomere maintenance genes that are associated with non-small cell lung cancer (NSCLC) risk [3]. Understanding the role of these genetic variations and their relationship with telomere dynamics could provide valuable insights into the pathogenesis of NSCLC and inform the development of novel diagnostic and therapeutic strategies.Telomeres are critical for maintaining chromosomal integrity and stability. They comprise tandem hexanucleotide repeats (TTAGGG) at the ends of eukaryotic chromosomes and terminate in a 3' single-strand guanine overhang [4]. The length of telomeres is regulated by telomere maintenance genes, which primarily belong to three complexes: Shelterin, CTC1–STN1–TEN1 (CST), and Telomerase [5–7]. Several case–control association studies have reported an association between shortened telomere length and lung cancer risk [8–10].

**Fig. 1:**
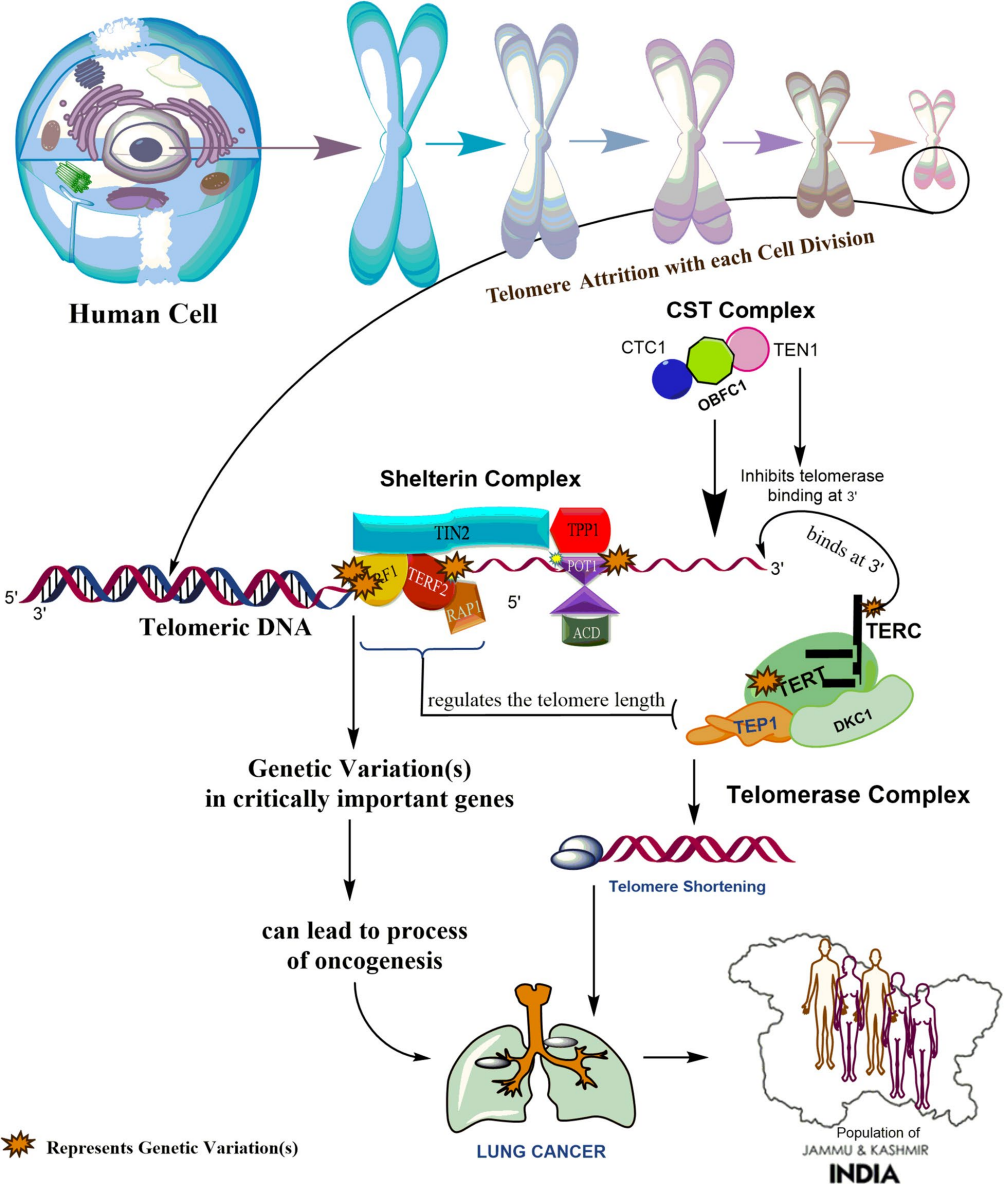
Illustration of genes vital for the maintenance of telomere ends and the regulation of telomere length. The genetic variants targeted from the telomere maintenance genes in the study are designated by (*) asterisk mark like rs10069690 of *TERT*, rs10228682 of *POT1,* rs251796 and rs2975843 of *TERF2* of shelterin complex

The Shelterin complex is composed of six integral genes: Telomeric Repeat Binding Factor 1 (TERF1), Telomeric Repeat Binding Factor 2 (TERF2), Protection of Telomeres 1 (POT1), TERF1 Interacting Nuclear Factor 2 (TIN2), Tripeptidyl Peptidase 1 (TPP1), and Repressor/activator protein 1 (RAP1) (Fig. 1). The Shelterin complex, along with the CST and Telomerase complexes, play a critical role in preserving telomere integrity [11, 12]. Emerging evidence suggests that genetic variants in these complexes may contribute to telomere dysfunction, which has been implicated in cancer development and progression [13, 14]. For instance, aberrant telomere lengthening, due to telomerase reactivation, has been observed in approximately 90% of human cancers, including NSCLC [15–17]. Conversely, telomere shortening has been associated with increased chromosomal instability, which may contribute to the initiation and progression of malignancies [18].

In the context of lung cancer, previous studies have reported associations between genetic variations in TERT and the risk of lung cancer [7, 19]. TERT encodes the catalytic subunit of the telomerase enzyme, which plays a crucial role in maintaining telomere length and protecting against telomere attrition [20]. Moreover, POT1 and TERF2, other components of the Shelterin complex, have also been implicated in lung cancer susceptibility [21, 22]. POT1 is involved in the regulation of telomere length and protection against telomere dysfunction-induced DNA damage, while TERF2 is responsible for the stabilization of the telomeric loop structure and the prevention of DNA damage response activation [23, 24].

Besides genetic variations, epigenetic modifications and environmental factors, such as smoking and exposure to air pollution, have also been shown to influence telomere length and contribute to lung cancer risk [25, 26]. For example, oxidative stress from cigarette smoke exposure can lead to DNA damage and telomere attrition, which may subsequently increase the risk of lung cancer [27]. Therefore, understanding the complex interplay between genetic, epigenetic, and environmental factors in telomere length regulation is crucial for comprehending the pathogenesis of lung cancer and identifying potential targets for intervention.

The present study targets the variants rs10069690, rs10228682, rs251796, and rs2975843 in genes – TERT, POT1, and TERF2, respectively, from the shelterin complex among 723 individuals (162 non-small cell lung cancer cases and 561 healthy control) in the ethnic population of Jammu and Kashmir, North India for NSCLC risk. Furthermore, the telomere length variation among 216 individuals (108 non-small cell lung cancer cases and 108 healthy controls) in the ethnic population of Jammu and Kashmir, North India, was explored using a dual labeled fluorescence probe-based assay [28]. Moreover, another subset of samples of the same population group was evaluated for NSCLC risk, targeting shelterin complex genes by Taqman probe-based methodology.

In summary, this study aims to provide a comprehensive investigation of the associations between genetic variants in telomere maintenance genes and NSCLC risk in the Jammu and Kashmir population of North India. By examining the potential role of telomere length as a prognostic biomarker for NSCLC diagnosis, we hope to contribute valuable insights to the growing body of knowledge on lung cancer etiology and inform the development of novel diagnostic and therapeutic strategies.

## Materials & methods

### Ethical statement

The study had been approved by the Institutional Ethics Review Board (IERB) of Shri Mata Vaishno Devi University vide IERB Serial No: SMVDU/IERB/16/41. Written informed consent was acquired from all the participants enrolled in the present study.

### Sampling

Seven hundred twenty-three individuals (162 non-small cell lung cancer cases and 561 healthy control) recruited for the study were evaluated by MassARRAY, and TaqMan Genotyping evaluated 254 non-small cell lung cancer cases and 405 healthy controls. Furthermore, we also examined the telomere length variation among 216 individuals (108 non-small cell lung cancer cases and 108 healthy controls) after approval from the Institutional Ethical Review Board (IERB). All cancer cases were histopathologically confirmed. Two milliliters of venous blood samples were collected from each participant in an EDTA vial.

### DNA isolation

FlexiGene® DNA kit (Qiagen, catalog No. 51206) method was used to extract genomic DNA from the blood samples. The quantity and quality control analysis of genomic DNA was performed by carrying out UV spectrophotometer (Eppendorf Biospectrometer®, Hamburg, Germany) analysis and Gel electrophoresis, respectively. The purified DNA was kept at 4ºC till further use at a concentration of 10 ng/µl.

### Selection of variants and genotyping

This study selected the genetic variants of telomere maintenance genes, which have a critical role in non-small cell lung cancer through GWAS and replication studies using the candidate gene approach (CGA)). Finally, 4 genetic variants of 3 vital genes were shortlisted. The details of genetic variants are discussed in Supplementary Table 1. Genotyping was performed on a high-throughput Agena Mass ARRAY platform and Taqman genotyping using Taqman Chemistry. To carry out genotyping, the central Mass ARRAY Analyzer facility at Shri Mata Vaishno Devi University was used, and recommended protocol(s) were followed. AgenaCxV.2.0 was used to design forward, reverse, and single base extension primers (customized) for MassARRAY (Supplementary Table 2). Sequenom Typer 4.0. Software was used to analyze genotype calls. In order to exclude the call errors via spectrograms, all genotype calls were cross-checked. The subjects were excluded from the study if the missing genotypes were higher than 10%. Those that don’t follow the Hardy–Weinberg Equilibrium (HWE) (*p*-value < 0.05) were also omitted from the study. The genotyping results were replicated in 10% of random samples, and the concordance rate was 98.5%. In the reaction of 384 well plates, one positive and one negative control were added for quality check. Mass ARRAY Cluster Plots were obtained after genotyping.**.** Another high throughput technique, TaqMan genotyping using TaqMan Chemistry, was used to evaluate the genetic association of rs10069690, rs2242652 of *TERT* & rs251796 of *TERF2* with non-small cell lung cancer risk. Moreover, Leucocyte Telomere Length was assessed by using dual labeled fluorescence probe-based assay. The data was exported in Excel format from the MxPro Software. The triplicates were averaged, and then the averaged Ct (Threshold) value was used to calculate relative telomere length (RTL). RTL was calculated based on Ct values, as [2Ct (telomeres)/2Ct (scg)]–1 = 2^–ΔCt^ [28].

### Statistical analysis

A statistical t-test was used to compare the clinical characteristics between cases and controls. Genotype data were analyzed using PLINK v. 1.07 [29] and IBM SPSS statistics 20 software [30]. All the genetic variants were tested for Hardy–Weinberg equilibrium using the chi-square test. The association of variants with non-small lung cancer risk was validated by binary logistic regression analysis adjusted for confounding factors like age, gender, and Body Mass Index (BMI). The odds ratios (ORs) were calculated based on the risk allele observed in this study. One-way ANOVA was employed to compare the clinical characteristics of different genotypes for each variant, adjusted for age and gender (Supplementary table 3). To estimate telomere length student t-test was used to check the significant difference between cases and controls.

### Potential role of the variants

Expression Quantitative Trait Loci (eQTL), UCSC Genome Browser, Encyclopedia of DNA Elements (ENCODE) (V3), and HaploReg v4.1 database [31, 32] tools were employed for analyzing the transcriptional regulatory role like histone modifications, DNase hypersensitivity and binding sites for the transcription factor. Besides that, the effect of the variant on splicing was evaluated by using the web tool Human Spicing Finder (HSF) 3.1 and ESE Finder (3.0) [33, 34].

## Results

The present study targeted the genetic variations in telomere maintenance genes like *TERT, POT1,* and *TERF2* in the ethnic population of Jammu and Kashmir, North India, for NSCLC risk using MassARRAY.With this perspective, the association between genetic variant rs10069690 of *TERT* (Telomere Reverse Transcriptase), rs10228682 of *POT1* (Protection of Telomeres), rs251796, rs2975843 of *TERF2* (Telomere Repeating Factor 2) and non-small cell lung cancer was evaluated. It was observed that rs10069690 of *TERT* was significantly associated with non-small lung cancer risk with an odds ratio = 1.65 (1.08–2.52 at 95% CI);* p-*value *(*adjusted) = 0.019, as shown in Table 1. Genetic variant rs10228682 of *POT1* was also found to be significantly associated with non-small cell lung cancer risk with an odds ratio = 1.88 (1.25–2.83 at 95% of CI);* p-*value *(*adjusted) = 0.002 as shown in Table 1. The study also targeted the genetic variations in telomere maintenance genes, rs10069690 & rs2242652 of *TERT* using TaqMan probe-based genotyping on the same population group. It was observed that rs10069690 & rs2242652 were found to be significantly associated with NSCLC risk, with an odds Ratio (OR) of 1.47 (1.06–2.05 at 95% CI); *p-*value (adjusted) = 0.02; 1.65 (1.18–2.32 at 95% CI); *p-*value (adjusted) = 0.003 respectively as shown in Table 2.

**Table 1:** Allelic, Genotypic distribution and logistic regression analysis of significant variants of genes in our study using the MassARRAY platform

Variant	rs10228682		rs10069690	
**Nearest gene w.r.t variant**	***POT1***		***TERT***	
**Polymorphism**	**C/T**		**C/T**	
**Ancestral allele**	**C**		**C**	
**Allele distribution**	**C**	**T**	**C**	**T**
**Cases**	0.625	0.705	0.866	0.817
**Controls**	0.375	0.295	0.134	0.183
**Odds ratio at 95% CI**	1.43 (1.09–1.88)		0.7(0.49–1.00)	
**Total HWE**	0.111		0.951	
**Genotypic model**	**Dominant**		**Recessive**	
	**(CC + CT vs TT)**		**(CC vs TT + CT)**	
*p*-Value*	0.002		0.019	
**Odds ratio at 95% CI**	1.88 (1.25–2.83)		1.65 (1.08–2.52)	

^*^Adjusted for Age, Gender, and BMI

**Table 2:** Allelic, Genotypic distribution and logistic regression analysis of significant variants of genes in our study using Taqman Chemistry

Variant	rs10069690		rs2242652	
**Nearest gene w.r.t variant**	***TERT***		***TERT***	
**Polymorphism**	**C/T**		**A/G**	
**Ancestral allele**	**C**		**A**	
**Allele distribution**	**C**	**T**	**A**	**G**
**Cases**	**0.67**	**0.33**	**0.74**	**0.26**
**Controls**	**0.73**	**0.27**	**0.81**	**0.19**
**Odds ratio at 95% CI**	**1.29 (1.01–1.64)**		**1.46 (1.12–1.89)**	
**Total HWE**	**0.67**		**0.48**	
**Genotypic model**	**Dominant model**		**Dominant model**	
	**TT/CT vs CC**		**GG/AG vs AA**	
*p*-Value*	0.02		0.003	
**Odds ratio at 95% CI**	1.47 (1.06–2.05)		1.65 (1.18–2.32)	

*Adjusted for Age, Gender, and BMI

Furthermore, we deemed it essential to assess the results concerning adenocarcinoma and squamous cell carcinoma independently. This distinction stems from the inherent heterogeneity of NSCLC, recognizing that varied underlying biological mechanisms and risk factors may govern divergent subtypes. Our observations pointed towards a meaningful association between the polymorphisms rs10069690 and rs2242652 in both Adenocarcinoma and Squamous cell carcinoma subtypes and the prevalence of non-small cell lung cancer within the population of Jammu & Kashmir, North India. The statistical significance of these associations is underscored by *p*-values of 0.04 and 0.002 for Adenocarcinoma and 0.046 and 0.002 for Squamous cell carcinoma, respectively. These findings are illustrated in detail in Supplementary Table 4.

However, the variants rs251796 and rs2975843 of *TERF2* were evaluated using the MassARRAY platform and Taqman genotyping. No statistically significant association was observed with non-small cell lung cancer risk in the studied population with an odds ratio = 0.82 (0.62–1.08 at 95% of CI);* p-*value = 0.16; Odds ratio = 0.90 (0.70–1.17 at 95% of CI);* p-*value = 0.46 (MassARRAY Platform); Odds ratio (OR) = 0.94 (0.75–1.19 at 95% CI); *p*-value = 0.61 (Taqman Chemistry) respectively as shown in Table 3. Moreover, exploring the bioinformatic approach, cis-eQTL analysis suggested that risk allele (T) of rs10228682 of *POT1* is linked with upregulation of the expression of the gene in lungs (*p*-value = 2.2E-18 and normalized effect size (NES) = 0.45). Since the gene is critical in telomere maintenance, the upregulation of the gene might affect telomere physiology.

**Table 3:** Allelic and Genotypic distribution of the variants, which did not show significant association with NSCLC in population of J&K, North India using MassARRAY and Taqman Chemistry

Variant	rs251796		rs2975843		rs251796	
Genotyping technique	**MassARRAY**		**MassARRAY**		**Taqman Chemistry**	
Nearest gene W.R.T variant	***TERF2***		***TERF2***		***TERF2***	
Polymorphism	**A/G**		**A/G**		**A/G**	
Ancestral allele	**A**		**T**		**A**	
Allele distribution	**A**	**G**	**A**	**G**	**A**	**G**
Cases	**0.70**	**0.30**	**0.58**	**0.42**	**0.68**	**0.32**
Controls	**0.66**	**0.34**	**0.56**	**0.44**	**0.66**	**0.34**
Odds ratio at 95% CI	**0.82** (0.62–1.08)		**0.90** (0.70–1.17)		**0.94** (0.75–1.19)	
*p* Value	**0.1623**		**0.4685**		**0.617**	
Total HWE	0.629		0.126		0.921	

Besides that, to examine the consequence of this genetic variant on *POT1,* an in silico approach by Human Splicing Finder (HSF) and exonic splicing enhancers (ESE) was explored. The widely used algorithms for predicting enhancer/silencer motifs by HSF demonstrated that rs10228682 results in a broken site for 9G8. Mutant Motif Value = 79.8 (Reference value 59.24) (Table 4).

**Table 4:** Putative Role of the associated variants with non-small cell lung cancer in J&K, North- India utilizing the information from the various databases, including GTEX, UCSC genome browser and HSF

Variant	Allele Ref/Alt	eQTL gene	eQTL Tissue	eQTL sample size	eQTL NES	eQTL p-value	eQTL m-value	Putative role (cis- eQTL) of variant	Regulatory role of variant (ENCODE and Haploreg data)	Splicing effect
rs10228682	C/T*	***POT1-AS1***	Lung	515	0.45	2.2E-18	1	Significant and Up regulation	NA	Broken site for 9G8
rs10069690	*T/C	***TERT***	Lung	NA	NA	NA	NA	NA	NA	Creation of new site/ broken site for SF2/ASF (IgM/BRCA1), SF2/ASF and SRp40
rs251796	*A/G	***TERF2***	Lung	515	-0.470	1.3E-19	1	Significant and down regulation	NA	NA
rs2975843	*A/G	***TERF2***	Lung	515	-0.067	0.07	0.988	Down regulated and non-significant	H3K4me3_Pro, H3K4me1_Enh	NA

^*^represents risk allele in this study, NES Normalized Effect Size in Eqtl, *m-value* Posterior probability that effect exists in each tissue, ranges between 0 and 1, *ESE* Exonic Splicing Enhancers, *SR* Serine-Arginine rich proteins, *9G8*, *SC35* SR splicing factor, *SF2/ASF* (IgM-BRCA1) Serine-Arginine rich proteins. *H3K4me3* Chemical modification (methylation) of the histone proteins (H3) at lysine 4, marks promoters that are active or poised to be activated, Enh Enhancers, *Pro* Promoters

**Fig. 2:**
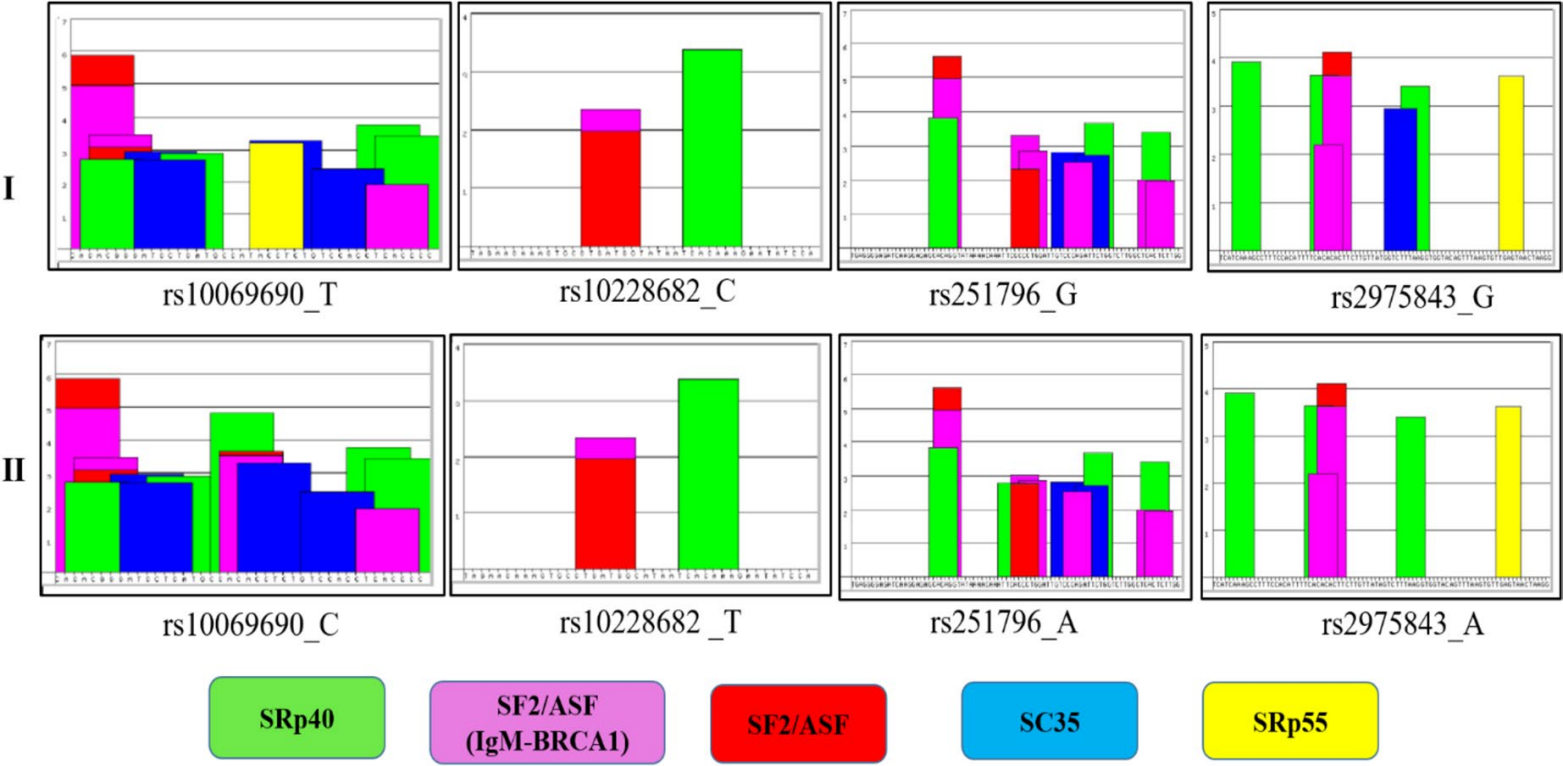
Effect of genetic variation on the Exonic Splicing Enhancers (ESEs) according to the ESE prediction tool. ESE finder enables the recognition of potential ESE sites. The elevation of the colored bars represents the motif scores, and the bars' circumference indicates the motif's length. Bars in red, yellow, blue, purple, and green indicate potential binding sites for Serine-Arginine (SR) proteins SF2/ASF, SRp55, SC35, SF2/ASF (IgM-BRCA1) and SRp40, respectively. Panel-I signifies the ESE sequence with the allele not posing a risk in the population under study, and panel –II denotes the ESE sequence with the risk allele in the studied population

Additionally, an assessment of the impact of the genetic variant rs10069690 on the gene through a computational analysis by Human Splicing Finder (HSF) was carried out. The study revealed a disruption in the exonic splicing enhancer (ESE) site. Most of the algorithms employed by the HSF (v.3.1) tool for the prediction of enhancer/silencer motifs, as illustrated in Table 4 and Fig. 2, suggested that rs10069690 leads to the formation of new binding sites for the (serine/arginine-rich protein) SR40(90.90), SF2/ASF (82.99), and SF2/ASF (IgM-BRCA1) (83.31). The results showed that modifications in the binding of the splicing factor of the exonic splicing enhancer (ESE) intronic site hint at its influence on splice site binding. Moreover, cis-eQTL analysis implied that the risk allele (A) of rs2251796 of TERF2 is associated with a decrease in the gene's expression in the lungs (*p*-value = 1.3E-19 and normalized effect size (NES) =—0.470). Given the gene's critical role in maintaining telomeres, this reduced expression may influence telomere physiology. Simultaneously, the risk allele (A) of rs2975843 of TERF2 is linked with a decrease in the regulation of gene expression in the lungs, with the site displaying histone marks like (H3K4me3_Pro, H3K4me1_Enh), suggesting involvement in epigenetic regulatory activities.

The relative telomere length in peripheral blood lymphocytes was measured by using dual labeled fluorescence probe-based assay in 108 non-small cell lung cancer patients and 108 healthy controls (triplicates). The cases and controls were frequency-matched for age, sex, alcohol, and smoking status. Telomere length was found to be significantly shorter in non-small cell lung cancer patients than in controls *(p-*value = 4 × 10^–3^*)*. In our quest for deeper insight, we sub-categorized the NSCLC dataset by categorizing it into distinct subtypes—AC, SCC, and LUAD. This meticulous subcategorization allowed us to investigate the association between telomere length and these respective NSCLC subtypes.

Our findings underscored a significant reduction in telomere length across all cancer subtypes compared to the control group. Specifically, patients with AC, SCC, and LUAD exhibited considerably shorter telomeres, backed by statistically significant p-values of 0.04002, 0.0367, and 0.00042, respectively. This sheds new light on the intricate dynamics between different NSCLC subtypes and telomere attrition.

The regression analysis was performed using the IBM SPSS Statistical software tool (V-20), which showed the effect of covariates on telomere length when adjusted with age, gender, alcohol, smoking status, and gutkha intake. It was observed that smoking is significantly associated with telomere attrition with an Odds ratio = 3.48 (1.7–7.16 at 95% of CI);* p-*value = 001 (Tables 5 and 6).

**Table 5:** Logistic regression analysis adjusted with Age, Gender and BMI

Variables	*p*-value**	95% CI
TL^a^	0.002	2.31(1.90–3.74)
Smoking	0.01	3.41(1.70–6.10)
Alchol intake	0.04	0.93 (0.40–1.90)
Guthka intake	0.04	0.13 (0.03–1.02)

^a^Telomere Length; **Age, Gender and BMI

**Table 6:** Logistic regression analysis adjusted with Age, Gender and BMI

Variables	*p*-value*	95% CI
Smoking	0.02	3.90(1.8–6.3)
Alchol intake	0.05	0.86 (0.6–2.2)
Guthka intake	0.48	1.70(1.0–3.02)

^*^Age, Gender and BMI

## Discussion and conclusion

The success of identifying many genetic variants and susceptibility loci in critically important genes was achieved through genome-wide association studies (GWAS) and transcriptome-wide association studies (TWAS). Worldwide, more than 60 loci have been linked with lung cancer by GWAS and the candidate gene approach. These genes are linked with diverse pathways regulating cell growth, cell survival, apoptosis, and telomere attrition.

Therefore, the present study explored the association between telomere-associated pathway genes *(TERF1, TERF2, POT1, TERT)* (Fig. 1) and non-small cell lung cancer risk in the less genetically explored population group.

So, we targeted to screen genetic variations in these genes for non-small cell lung cancer risk in an ethnic population of Jammu and Kashmir, North India. We previously conducted a case–control association study of genetic variant rs2853677 of *TERT* on susceptibility to non-small cell lung Carcinoma in the population of Jammu and Kashmir (*p* = 0.03) [7].

The present study observed that rs10069690 of *TERT* was significantly associated with non-small lung cancer risk using the MassARRAY platform with an Odds ratio = 1.65 (1.08–2.52 at 95% CI);* p-*value *(*adjusted) = 0.019. Moreover, rs10069690 & rs2242652 of *TERT* evaluated using Taqman-based genotyping were also found to be significantly associated with NSCLC risk, with an odds Ratio (OR) of 1.47 (1.06–2.05 at 95% CI); *p-*value (adjusted) = 0.02; 1.65 (1.18–2.32 at 95% CI); *p-*value (adjusted) = 0.003 respectively. These findings are consistent with the lung cancer risk in the Chinese Han population [35, 36]. Furthermore, genetic variant rs10228682 of *POT1* evaluated by MassARRAY was also found to be significantly associated with non-small cell lung cancer risk with an Odds ratio = 1.88 (1.25–2.83 at 95% CI);* p-*value (adjusted) = 0.002. These observations have been associated with Chinese lung cancer risk [37]. However, genetic variants rs251796 and rs2975843 of *TERF2* did not significantly affect non-small cell lung cancer risk with* p* value = 0.162, 0.61 & 0.468, respectively.

Furthermore, to evaluate the effect of these polymorphisms on the physiology of the telomere maintenance gene, the bioinformatic in silico analysis of these variants was performed by (eQTL), UCSC, ENCODE (V3), HaploReg v4.1, (HSF) 3.1and ESE finder (3.0). It was observed that either some are involved in epigenetic regulation, some result in broken sites, and some result in the creation of new sites, which eventually have a significant effect on the physiology of shelterin complex and telomere length regulation. Moreover, the splicing/epigenetic effect of the variants needs to be confirmed in in vitro analysis. The main aim of identifying these associations in telomere mantainence genes using dual high throughput techniques was to understand the genetic heterogeneity of non-small cell lung cancer and the possibility of using the identified genetic variant of telomere maintenance gene *(TERT, POT1, TERF2)* as a prognostic biomarker for diagnosis of non-small cell lung cancer.

The limitations of the present case–control association study include the relatively small sample size, and secondly, more data is needed on how the actual genetic polymorphic mechanism of the *TERT, TERF2* and *POTI* genes, which remains conserved among species and different ethnic groups contributes to lung cancer risk in the J&K population, North India. Moreover, we also quest to evaluate these variants with large sample sizes in different cancers in the future with functional validations.

### Supplementary Information

**Additional file 1:** **Supplementary Table 1.** Details of the variants selected for the study of non-small cell lung cancer in the Jammu and Kashmir population.

**Additional file 2:** **Supplementary Table 2.** List of variants and their primer and probe (UEP) sequence.

**Additional file 3:** **Supplementary Table 3.** One-way ANOVA of significant Variants with quantitative traits. 

**Additional file 4:** **Supplementary Table 4.** Allelic and Genotypic distribution of the variants and their association with two main subtypes of NSCLC.

## Data Availability

The datasets generated or analyzed during the current study are not publicly available but are available with the corresponding author and can be provided upon reasonable request.
