# Supplementary material for: Associations between telomere attrition, genetic variants in telomere maintenance genes, and non-small cell lung cancer risk in the Jammu and Kashmir population of North India
Source: BMC Cancer. 2023 Sep 18;23:874. doi: 10.1186/s12885-023-11387-z (PMC10506276; doi:10.1186/s12885-023-11387-z)
Supplement: Supplementary file 1 — Additional file 1: Supplementary Table 1. Details of the variants selected for the study of non-small cell lung cancer in the Jammu and Kashmir population. [file 12885_2023_11387_MOESM1_ESM.docx]

**Supplementary Table 1:** Details of the variants selected for the study of non-small cell lung cancer in the Jammu and Kashmir population.

| S. No. | SNP | Chromosome: position | Location of the Variant w.r.t Gene | Function of Gene |
| --- | --- | --- | --- | --- |
|  |  |  |  |  |
| 1. | rs10069690 | 5:1279675 | Intron Variant | Telomerase is a ribonucleoprotein polymerase that maintains telomere ends by adding the telomere repeat TTAGGG. The enzyme consists of a protein component with reverse transcriptase activity, encoded by this gene, and an RNA component which serves as a template for the telomere repeat. |
| 2. | rs10228682 | 7:124897982 | Intron Variant | This gene is a member of the telomere family and encodes a nuclear protein involved in telomere maintenance. Specifically, this protein functions as a member of a multi-protein complex that binds to the TTAGGG repeats of telomeres, regulating telomere length and protecting chromosome ends from atypical recombination, catastrophic chromosome instability, and abnormal chromosome segregation. |
| 3.  4. | rs251796 | 16:69361531 | Intron Variant | TERF2 is an integral member of shelterin nucleo protein complex and negative regulator of the telomere length. It is the protective function and protects the telomeres from the DNA damage and prevents the chromosome end fusion |
|  | rs2975843 | 8:73007753 | Upstream transcript variant |  |
