# Supplementary material for: Associations between telomere attrition, genetic variants in telomere maintenance genes, and non-small cell lung cancer risk in the Jammu and Kashmir population of North India
Source: BMC Cancer. 2023 Sep 18;23:874. doi: 10.1186/s12885-023-11387-z (PMC10506276; doi:10.1186/s12885-023-11387-z)
Supplement: Supplementary file 2 — Additional file 2: Supplementary Table 2. List of variants and their primer and probe (UEP) sequence. [file 12885_2023_11387_MOESM2_ESM.docx]

**Supplementary Table 2**. List of variants and their primer and probe (UEP) sequence.

| Variant | Gene | First Primer Sequence | Second Primer Sequence | UEP Sequence |
| --- | --- | --- | --- | --- |
| rs10069690 | ***TERT*** | ACGTTGGATGCTGTTTGAAACGGGTTCCTG | ACGTTGGATGTCATCTGAGGAGAGTGTGGG | gtttCACACGGGATCCTCATGCCA |
| rs10228682 | ***POT1*** | ACGTTGGATGATCAAGTAGAACAAAGTGCC | ACGTTGGATGACCGCACAGACATTCTAGAC | cttcGAACAAAGTGCCTGATGG |
| rs251796 | ***TERF2*** | ACGTTGGATGACTTGCCTTTGGGTACTCTG | ACGTTGGATGAGTGAGCCAAGACCAGAATC | TGGGACAATCCAGGG |
| rs2975843 | ***TERF2*** | ACGTTGGATGGTGCCTTAGTTACTCAACAC | ACGTTGGATGCATCCAGCATCATCAAAGCC | CATTTTCACACACTTCTTGTTAT |
