# Supplementary material for: Associations between telomere attrition, genetic variants in telomere maintenance genes, and non-small cell lung cancer risk in the Jammu and Kashmir population of North India
Source: BMC Cancer. 2023 Sep 18;23:874. doi: 10.1186/s12885-023-11387-z (PMC10506276; doi:10.1186/s12885-023-11387-z)
Supplement: Supplementary file 3 — Additional file 3: Supplementary Table 3. One-way ANOVA of significant Variants with quantitative traits. [file 12885_2023_11387_MOESM3_ESM.docx]

**Supplementary Table 3.** One-way ANOVA of significant Variants with quantitative traits.

| Variant | rs10069690 | | | rs10228682 | | |
| --- | --- | --- | --- | --- | --- | --- |
| Nearest gene | ***TERT*** | | | ***POT1*** | | |
|  | **MEAN±SE** | | | **MEAN±SE** | | |
| Genotype | **TT** | **CT** | **CC** | **TT** | **CT** | **CC** |
| BMI | 22.00  ±1.4 | 21.74  ±3.8 | 22.70  ±3.9 | 23.34  ±3.9 | 21.66  ±3.2 | 23.00  ±4.7 |
| *P*-value | **0.40** | | | **0.29** | | |
| Age of onset | 48.50  ±0.7 | 59.18  ±9.7 | 61.66  ±9.8 | 62.41  ±10.5 | 60.69  ±9.2 | 58.60  ±9.2 |
| *P*-value | **0.07** | | | **0.03** | | |
| Smoking Status | 20.00  ±7.0 | 22.08  ±8.5 | 22.38  ±10.0 | 22.52  ±10.6 | 22.07  ±8.9 | 21.83  ±9.0 |
| P-value | **0.93** | | | **0.95** | | |
| Alcoholic Status | 9.50  ±7.7 | 11.50  ±7.2 | 14.17  ±7.6 | 15.33  ±7.7 | 12.21  ±7.2 | 9.83  ±7.6 |
| *P*-value | **0.34** | | | **0.41** | | |
