# Supplementary material for: Associations between telomere attrition, genetic variants in telomere maintenance genes, and non-small cell lung cancer risk in the Jammu and Kashmir population of North India
Source: BMC Cancer. 2023 Sep 18;23:874. doi: 10.1186/s12885-023-11387-z (PMC10506276; doi:10.1186/s12885-023-11387-z)
Supplement: Supplementary file 4 — Additional file 4: Supplementary Table 4. Allelic and Genotypic distribution of the variants and their association with two main subtypes of NSCLC. [file 12885_2023_11387_MOESM4_ESM.docx]

| Variant | rs10069690 | | | rs2242652 | | |
| --- | --- | --- | --- | --- | --- | --- |
| Nearest gene w.r.t. variant | *TERT* | | | *TERT* | | |
| Polymorphism | C/T | | | A/G | | |
| Ancestral Allele | C | | | A | | |
|  | **Adenocarcinoma** | | |  | | |
| Allele Distribution  Cases  Controls | C  0.67  0.73 | | T  0.33  0.27 | A  0.76  0.81 | G  0.24  0.19 | |
| Odds Ratio at 95% CI | 1.47 (1.00-2.14) | | | 1.57 (1.07-2.31) | | |
| Total HWE | 0.39 | | | 0.44 | | |
| Genotypic Model | Dominant model  TT/CT vs CC | | | Dominant model  GG/AG vs AA | | |
| *^*^P-*value | 0.048 | | | 0.002 | | |
|  | **Squamous Cell Carcinoma** | | |  | | |
| Allele Distribution  Cases  Controls | C  0.66  0.73 | T  0.34  0.27 | | A  0.72  0.81 | | G  0.28  0.19 |
| Odds Ratio at 95% CI | 1.53 (1.01-2.33) | | | 1.90 (1.25-2.91) | | |
| Total HWE | 0.29 | | | 0.13 | | |
| Genotypic Model | Dominant model  TT/CT vs CC | | | Dominant model  GG/AG vs AA | | |
| *^*^P-*value | 0.046 | | | 0.002 | | |

**Supplementary Table 4:** Allelic and Genotypic distribution of the variants and their association with two main subtypes of NSCLC.
